# Screening of GO-coated microporous polymeric filters for efficient paraquat removal: effect of support surface on membrane roughness and flux stability

**DOI:** 10.1039/d6ra00505e

**Published:** 2026-03-20

**Authors:** Syed Sibt-e-Hassan, Nurmeen Adil, Yan Wang, Syed Ghulam Musharraf

**Affiliations:** a HEJ Research Institute of Chemistry, International Center for Chemical and Biological Sciences, University of Karachi Karachi-75270 Pakistan musharraf1977@yahoo.com musharraf@iccs.edu (+92-21) 4819018-9 +92 21 34824924-5; 34819010; b Swedish Metabolomics Centre, Swedish University of Agricultural Sciences Umeå Sweden; c School of Chemistry and Pharmaceutical Sciences, Guangxi Normal University Guilin-541004 China

## Abstract

We report the fabrication and systematic evaluation of three thin-layer graphene oxide (GO) composite membranes prepared by vacuum-filtering a GO dispersion (nominal loading 0.42 mg cm^−2^) onto low-cost microporous supports (mixed cellulose ester, nylon, PVDF; 0.45 µm pore, 12 cm^2^). The membranes (M-GO, N-GO, P-GO) were characterized by AFM, SEM, XPS, and contact angle measurements to reveal support-dependent GO morphology and surface chemistry. At low (0.2 bar) transmembrane pressure (TMP), M-GO exhibited the highest steady-state water flux (425 ± 10 L m^−2^ h^−1^, *n* = 3), followed by N-GO and P-GO, while all GO-coated membranes achieved near-complete paraquat rejection (≤ LOD = 0.04 ppm) for feed concentrations of 0.1–1.0 ppm. Reusability tests on M-GO demonstrated ≥95% removal over five consecutive 1 h cycles with a flux recovery ratio (FRR) ≥ 65% after hydraulic flushing. In a 42 h continuous stability test at 0.2 bar, M-GO retained 66% of its initial flux and maintained ≥ 99% paraquat rejection. Tests in a simulated agricultural matrix (paraquat 5 ppm, 100 mM NaCl, 10 ppm humic acid) show a moderate flux decline (stabilizing at ∼55–60% of initial flux) with paraquat rejection > 90%, indicating robustness to ionic strength and natural organic matter. The head-to-head comparison isolates the decisive role of support surface roughness and porosity in governing GO layer formation, flux stability, and antifouling behavior, a pathway to low-pressure, high-flux membranes for cationic pesticide removal.

## Abstract 2

We report the fabrication and systematic evaluation of three thin-layer graphene oxide (GO) composite membranes prepared by vacuum-filtering a GO dispersion onto low-cost microporous supports (*i.e.* mixed cellulose ester, nylon, and PVDF).

## Graphical abstract

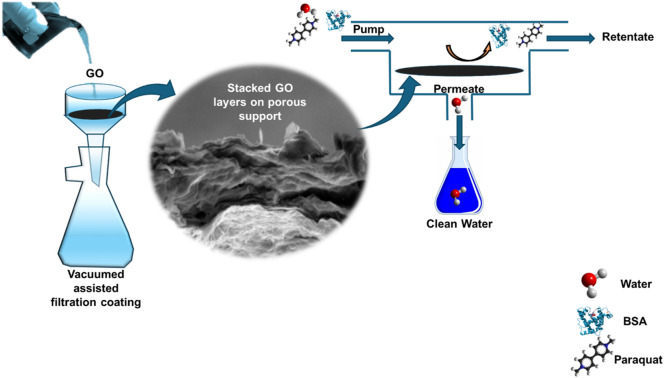

## 1 Introduction

Agricultural productivity is crucial for ensuring food security and economic stability, particularly in the face of a growing global population.^1^ Before the 1940s, weed control methods were largely mechanical and inefficient.^2^ The introduction of chemical herbicides in the 20th century revolutionized weed management in agricultural and non-agricultural settings.^3^ Paraquat (1,1′-dimethyl-4,4′-bipyridinium dichloride), a non-selective herbicide introduced in 1962, became widely adopted due to its high efficacy and affordability, effectively killing green plant tissue upon contact.^4^ However, its extensive use has led to contamination risks for non-target resources.^5^ Despite paraquat's inactivation through photodegradation and adsorption on clays or organic matter,^6^ its presence has been detected in drinking water sources, thanks to its high water solubility,^7^ posing a significant threat to human health due to its acute toxicity and links to diseases such as Parkinson's.^8,9^ Therefore, paraquat concentration levels in surface water must be carefully monitored and controlled according to the WHO acceptable daily intake (ADI) and the Environmental Protection Agency (EPA) reference dose (RfD) of 0.0045 mg kg^−1^ day^−1^, which translates to 90 µg L^−1^ (0.09 ppm) with daily consumption of 3 liters of drinking water, or 0.27 ppm.^5,10^ Canadian Drinking Water Quality Guidelines, however, set the maximum tolerable level of drinking water to 0.01 ppm (0.1 µg L^−1^).^11^

The need for cost-effective and efficient materials to remove paraquat from water, a primary exposure route to humans, is increasingly pressing.^12^ Conventional removal methods, including adsorption,^13^ ion exchange,^14^ photodegradation,^15^ biodegradation,^16^ and coagulation–flocculation,^17^ are often expensive, less effective, and require specialized equipment or personnel. Alternative methods, such as membrane-based separations (reverse osmosis and nano-filtration), suffer from severe fouling issues.^18^

Although activated carbon and carbon black are widely used as low-cost adsorbents for paraquat, they typically require separate chemical activation to introduce surface functionalities and lack the two-dimensional platelet geometry needed for uniform, tunable nanochannels on a membrane surface. In contrast, graphene oxide (GO) inherently bears a high density of oxygen-containing groups and can be deposited as a monolayer-to-few-layer film on polymeric supports, forming well-defined nanochannels that facilitate size-exclusion and electrostatic binding of cationic paraquat.^19^ Because only a thin GO coating is needed, the material cost per filter remains competitive with activated carbon, while delivering higher adsorption capacities and superior fouling resistance.

Recent studies have demonstrated that graphene-based materials possess exceptional adsorptive properties, making them highly effective for paraquat removal through adsorption.^20^ However, scaling up this removal method poses challenges. To address this, scalable technologies like membrane-based separations can be integrated with graphene-based materials. This approach enhances removal performance while maintaining scalability, as graphene-based composite membranes have also shown resistance to fouling.^21^

Several recent studies have demonstrated that coating microporous polymer membranes such as nylon, PVDF, and cellulose derivatives with graphene oxide (GO) significantly improves the removal of pesticides and related organic pollutants. For instance, in one study, GO-coated nylon membranes were prepared by vacuum filtering dilute GO dispersions (0.01 mg mL^−1^) onto 0.45 µm filters, achieving >90% dye rejection and up to 83% removal of phenolics like 2-naphthol.^22^ In another study, GO-chitosan-coated PVDF microfiltration membranes achieved >95% dye rejection and improved antifouling stability.^23^ Similarly, Mukherjee *et al.* (2018) applied GO-chitosan composites on ceramic ultrafiltration support, achieving >95% atrazine removal in a membrane bioreactor.^24^ These examples highlight the potential of GO-modified membranes for pesticide removal. However, most reports focus on dyes or phenolic pollutants and do not systematically compare multiple GO–polymer systems for paraquat.

Despite extensive work on free-standing GO membranes for water purification, few studies have systematically combined GO with low-cost, microporous polymeric supports to yield a truly scalable, mechanically robust composite. Despite the growing interest in graphene oxide (GO)-based membranes for water purification,^25^ systematic investigations isolating the role of the underlying support material remain limited. In many reported studies, variations in GO loading, fabrication conditions, or operating pressure obscure the intrinsic influence of support surface properties on membrane performance. As a result, the relationship between support morphology, GO deposition behavior, and the resulting flux–rejection trade-off is not yet fully understood, particularly under low-pressure conditions relevant to energy-efficient water treatment. This work's novelty is a controlled head-to-head comparison at identical GO mass loading (0.42 mg cm^−2^) across three low-cost microporous supports, revealing support-dependent GO morphology–flux–rejection relationships and enabling high-flux paraquat removal at very low pressure (0.2 bar).

## 2 Experimental

### 2.1. Chemicals and materials

All reagents and standards used were of HPLC grade. The following materials were obtained from Sigma Aldrich (USA): paraquat dichloride standard, acetonitrile, ammonium formate, formic acid, and bovine serum albumin (BSA, 98%). Graphite mesh was sourced from Millipore Merck (USA). Milli-Q water (MQW) was used throughout the study, obtained from the Milli-Q assembly (Millipore, USA). Commercial mixed cellulose ester (MCE) and nylon (N) membrane filters (0.45 µm pore size, 47 mm diameter) were purchased from Membrane Solutions (USA). Hydrophilic polyvinylidene fluoride (PVDF) membrane filters (0.45 µm pore size, 47 mm diameter) were obtained from Merck Millipore. Additionally, Sodium Chloride salt (NaCl, 99%) and humic acid were purchased from Sigma Aldrich.

All glassware was meticulously cleaned with acid, thoroughly washed with MQW, and dried in the oven before use to ensure cleanliness and prevent contamination.

### 2.2. Synthesis and characterization of GO

GO was synthesized following our previously reported procedure.^21^ For characterization, Fourier-transform infrared (FTIR) spectroscopy of the synthesized graphene oxide (GO) was conducted using a Bruker Vector 22 spectrometer, covering the mid-infrared range (400–4000 cm^−1^). Particle size distribution and zeta potential of the GO samples were analyzed through Dynamic Light Scattering (DLS) using a Nano-ZSP instrument (Malvern Instruments). These experiments were carried out at room temperature with disposable cuvettes for zeta size measurements and dip cell cuvettes for zeta potential, both at a scattering angle of 90°. X-ray photoelectron spectroscopy (XPS) was performed with a Kratos Axis Supra system employing monochromated Al Kα1 radiation (1486.6 eV). Survey scans were conducted over an analysis area of 300 × 700 microns using a slot collimator, with a pass energy of 160 eV. Region scans were executed with pass energies of 20 or 40 eV, and the full width at half maximum (FWHM) for the Ag 3d 5/2 peak at a pass energy of 40 eV was 0.71 eV. Data were processed using CasaXPS software. Morphological studies were carried out *via* scanning electron microscopy (SEM) using a Zeiss Crossbeam 350 SEMFIB-SEM instrument. X-ray diffraction (XRD) analysis was conducted using a Malvern PANalytical X'pert Powder instrument to determine the average interlayer spacing between GO flakes in the membranes.

### 2.3. Preparation of composite membranes

Composite graphene oxide membranes (GOMs) were fabricated *via* vacuum-assisted filtration at room temperature (25 °C) under approximately 1.0 bar vacuum. Commercial 0.45 µm membrane supports, *i.e.*, mixed cellulose ester (MCE), hydrophilic polyvinylidene fluoride (PVDF), and nylon, were used as substrates due to their uniform pore structure, hydrophilicity, and commercial availability.

GO coating dispersions were prepared by dispersing 5 mg of GO in 5 mL of deionized water (1 mg mL^−1^). The dispersion was bath-sonicated for 15 min to ensure homogeneous exfoliation, followed by an additional 5 min sonication immediately before coating to minimize aggregation and narrow the particle size distribution. The entire GO dispersion was then vacuum-filtered through each membrane support using a Buchner filtration setup. Filtration was continued until complete deposition of GO onto the membrane surface.

The effective membrane diameter was 3.9 cm, corresponding to an effective filtration area of 12 cm^2^. Since no detectable GO was observed in the filtrate (residual mass ≈ 0), the GO mass loading was calculated directly from the initial GO mass and membrane area, yielding a nominal loading of approximately 0.42 mg cm^−2^.

All coated membranes were vacuum-dried at 40 °C for 2 h to remove residual moisture. The resulting composite membranes were designated as M-GO, N-GO, and P-GO. For reproducibility, three independent membranes were prepared for each support under identical fabrication conditions.

### 2.4. Characterization of composite membranes

Atomic force microscopy (AFM) and SEM were employed to analyze the topography of the membranes. For SEM, both the pristine filters and the prepared composite membranes were coated with a gold film using a JEOL auto coater (JEF-1500). The membrane topography was then examined with an accelerated emission scanning electron microscope (JSM-6380-A) operated at 15 kV to capture detailed images.

The hydrophilicity of the membranes was evaluated by measuring the contact angles. The contact angles were measured at a neutral pH using ImageJ software by analyzing video frames captured exactly 3 seconds after the drop came into contact with the membrane surface. Contact-angle measurements were repeated on three independent spots per membrane (*n* = 3).

To evaluate the removal and permeance efficiency of each membrane, paraquat concentrations in the feed and permeate samples were quantified using an optimized LC-MS method.

More precisely, the permeation and rejection studies were carried out at 0.2 bar pressure. All the membranes were compacted for 20 min at 0.5 bar to reach a steady state before testing. Later, MQW-Flux, *J*_w_ (L m^−2^ h^−1^) was calculated using the equation below.
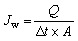
where ‘*Q*’ is the volume of permeated water (L), ‘*A*’ is the effective area of the membrane (m^2^), and Δ*t* is the permeation time (h).

The percentage rejection, ‘*R*’, was determined using the equation below.
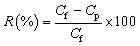
where ‘*C*_p_’ and ‘*C*_f_’ are the solute concentrations in permeate and feed, respectively.

Additionally, comprehensive experiments were conducted to investigate membrane properties such as porosity, water uptake capacity, and fouling resistance. Detailed results and discussion on water uptake/wettability and gravimetric porosity are provided in the SI.

The fouling resistivity of both membranes was tested by filtering a 300 ppm solution of BSA at a pH of 7 and room temperature with a TMP of 0.2 bar in the same crossflow filtration system. Initially, the pure MQW flux (*J*_w_1__) was measured for 1 h after reaching steady state. The feed was then replaced with the BSA solution, and the permeate flux (*J*_p_) was recorded for 1 h once steady state was achieved. After fouling, the membrane was cleaned by high-pressure forward flushing with MQW, and the recovered MQW flux (*J*_w_2__) was measured. The flux recovery ratio (FRR) was calculated from the MQW flux values to assess the antifouling performance of the membranes using the following equation.
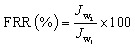


To gain a more thorough understanding of the fouling process, the degree of total flux drops due to total fouling (*R*_t_), reversible fouling (*R*_r_, fouling caused by concentration polarization), and irreversible fouling (*R*_ir_, fouling caused by the adsorption of protein molecules) were also studied using the following equations.
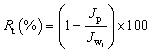

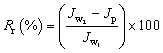

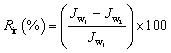


### 2.5. Paraquat-contaminated water samples

Initially, a stock solution of paraquat at 10 ppm was prepared by dissolving paraquat in MQW. This stock solution was subsequently diluted to obtain feed samples with concentrations of 1 ppm, 0.5 ppm, and 0.1 ppm. These feed samples were filtered through the developed membranes using our previously reported custom-made crossflow filtration method.^21^ A schematic illustration of the experimental setup is provided in Fig. S9 (SI). The permeate samples, collected after 1 h of steady-state filtration, were analyzed to determine the paraquat concentration. All measurements were performed in triplicate (*n* = 3) to ensure reproducibility.

Additional experiments were conducted using the best-performing membrane (*e.g.*, M-GO) to evaluate performance at higher paraquat concentrations (5–20 ppm) and in simulated agricultural water containing 5 ppm paraquat, NaCl (100 mM), and humic acid (10 ppm).

### 2.6. Quantification of paraquat from water samples using LC-ESI-QqQ-MS

Quantitative analysis was done by an Agilent 6460 Triple Quadrupole (QqQ) system with a jet stream ESI source (Agilent Technologies, Santa Clara, CA, USA).

The chromatographic analysis of paraquat was performed using an Agilent Zorbex Eclipse HILIC column (4.6 mm × 100 mm, 3.5 µm particle size) at a thermostat temperature of 45 °C. The chromatographic mobile phase A consisted of MQW with 50 mM ammonium formate and 0.5% formic acid. The mobile phase B was a mixture of MQW and acetonitrile (MQW : ACN, 25 : 75) with 50 mM ammonium formate and 0.5% formic acid. The mobile phase B gradient was ramped at a flow rate of 0.4 mL min^−1^ from 100% to 65% in 4.2 minutes, then ramped back to 100% in 4.3 minutes, and kept at 100% until 11 minutes.

A 10 µL sample of paraquat permeate was injected into the chromatographic system. The total run time of the analysis was 11 minutes. Positive ionization mode was used for the quantification of paraquat using mass spectrometry (MS). The MS parameters were set as follows: the scan range was 100–500 *m*/*z*, capillary voltage 4000 V, nebulizer gas pressure 40.0 psi, drying gas flow rate 8.0 L min^−1^ at 310.0 °C, fragmentor voltage 50.0 V, and collision energy 17.0 V.

To establish the calibration for quantification, a 10 ppm standard paraquat solution was diluted to concentrations of (0.05, 0.25, 0.5, 0.75, 1, 1.5, and 2 ppm), creating a seven-point calibration curve. The Limit of Detection (LOD) and Limit of Quantitation (LOQ) were calculated using the standard deviation (*σ*) and slope (*S*) of the calibration curve, according to the formulas: LOD = 3.3 *σ*/*S* and LOQ = 10 *σ*/*S*. Quantification was performed in triplicate (*n* = 3).

## 3 Results and discussion

### 3.1. Characterization of GO

As reported in our previous paper,^21^ the prepared GO was analyzed by FT-IR, X-ray Photoelectron Spectroscopy (XPS), Dynamic Light Scattering Spectroscopy (DLS), Zeta potential, and SEM the results are in the SI, see Fig. S1.

### 3.2. Characterization of GO composite membranes

After the coating process, the surface morphology and roughness of all the membranes were examined using AFM analysis.

**Fig. 1:**
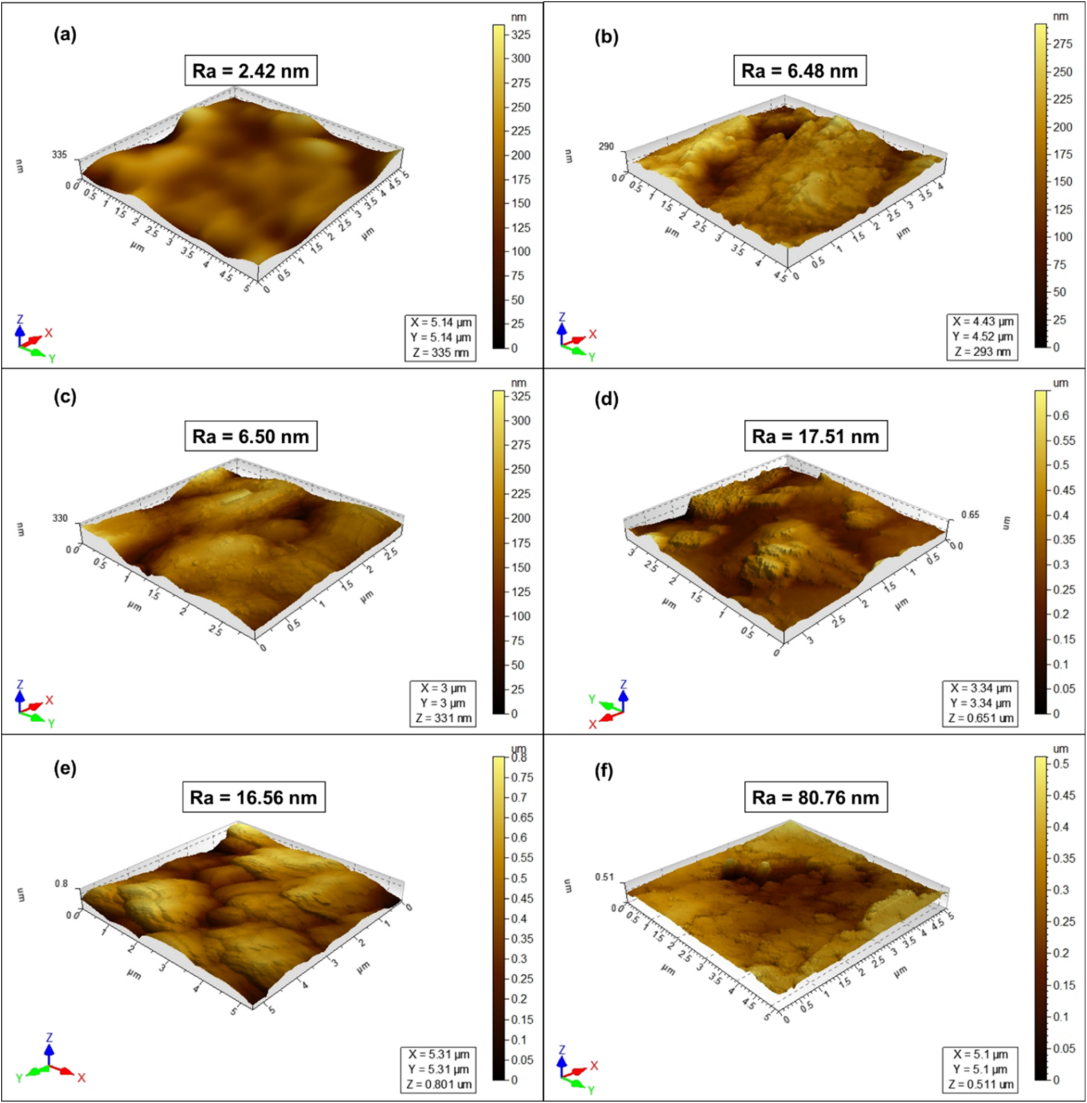
AFM surface analysis of all membranes. (a) and (b) show the surface roughness of the M and M-GO membranes, respectively; (c) and (d) correspond to the N and N-GO membranes; (e) and (f) represent the P and P-GO membranes.

The surface roughness of the underlying support membrane plays a pivotal role in determining the morphology and uniformity of the GO layer deposited *via* vacuum-assisted filtration. In our case, the supports, *i.e.*, M, N, and P, exhibit increasing roughness in the order M < N < P (see Fig. 1a, c, and e). This gradation in roughness likely leads to a corresponding increase in the surface area available for GO deposition, facilitating greater GO accumulation on rougher substrates. Studies have shown that increased surface roughness can enhance the adhesion and stability of nanomaterial coatings like GO due to mechanical interlocking and increased contact area.^26^ However, excessive roughness may also lead to non-uniform coatings, potentially introducing defects or variations in membrane performance.^27^

The augmented roughness post-GO coating suggests a thicker or more textured GO layer, which can influence both flux and surface area. While GO layers can enhance hydrophilicity and thus water permeability, overly thick or uneven coatings may impede flow, reducing flux.^28^ Increased roughness generally correlates with higher surface area, potentially providing more active sites for adsorption or interaction with target molecules like paraquat. Therefore, an optimal balance is crucial, *i.e.*, sufficient roughness to enhance surface area without compromising flux due to excessive thickness or irregularities.

**Fig. 2:**
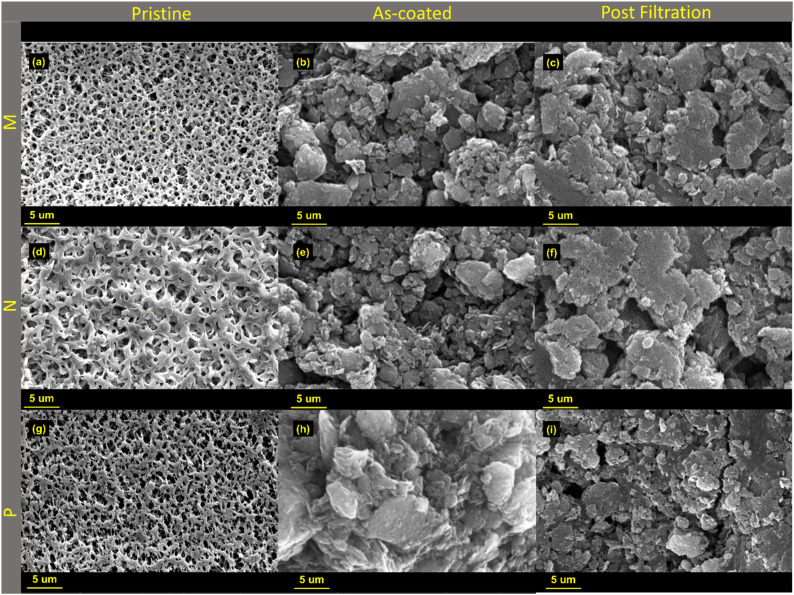
Top-view images of the selected membranes. (a) Shows pristine M, (b) shows M-GO after GO coating, and (c) shows M-GO after water filtration. (d) Shows pristine N, (e) shows N-GO after coating, and (f) shows N-GO post-filtration. (g) Shows pristine P, (h) shows P-GO after coating, and (i) shows P-GO following water filtration.

Building on the AFM-derived roughness hierarchy (M < N < P), the post-filtration SEMs (see Fig. 2) reveal clear morphological trends that mirror our surface-topography data. From Fig. 2b, we see a relatively thin, conformal GO film with few multilayer stacks in M-GO. The GO flakes lay nearly flush with the cellulose ester pores, producing a compact, uniform coating with minimal nodules. This morphology maximizes –OH/–COOH exposure consistent with the low contact angle and preserves high flux.

However, on the other hand, N-GO shows (see Fig. 2e) that GO flakes preferentially fill the deeper tortuous pores, creating small aggregates at valley bottoms and occasional overhangs at peaks. This partial mechanical interlocking enhances adhesion (good stability) but introduces mild heterogeneity, which correlates with a modest flux reduction relative to M-GO.

As far as P-GO is concerned, the SEM (Fig. 2h) displays thick, multilayer GO platelets bridging across microfilaments and forming irregular “islands”. These large agglomerates produce a highly textured surface that boosts areal coverage but at the expense of uniform pore channels and water pathways, explaining the observed drop in permeability.

In all three cases, the post-filtration SEMs demonstrate that GO fully coats the support, yet the degree of flake stacking and film uniformity scales directly with underlying roughness.

SEM of post-filtration (42 h) composite membranes (see Fig. 2c, f and i) shows more compacted and uniform morphology as compared to the as-prepared ones. The analysis revealed that the M-GO, N-GO, and P-GO composite membranes have completely covered the surface of the support material (Fig. 2). The same also applies in the case of N-GO, which we have reported in our previous study.^21^

**Fig. 3:**
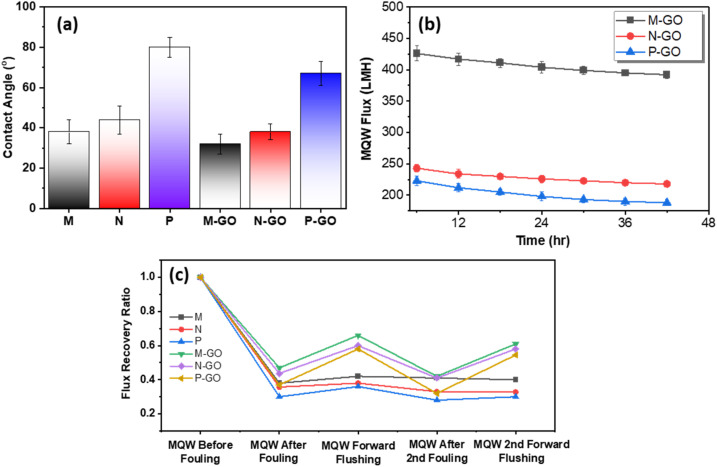
(a): Contact angle values at the third second after the drop touches the surface of all composite membranes under neutral pH conditions. (b): Steady-state flux profiles of all composite membranes and their respective porous supports during a 42 hours run at 0.2 bar. (c): Flux Recovery Ratio (FRR) of all composite membranes and their corresponding porous supports after two fouling and forward flushing cycles, normalized to the initial pure water flux (*J*_0_ = 1) measured before fouling.

As shown in Fig. 3a, the coating significantly enhanced the hydrophilicity of the new composite membrane, with the M-GO membrane exhibiting the lowest contact angle due to the high hydrophilicity of the coated GO plus the support. However, despite the expectation that all GO-coated surfaces should exhibit similar contact angles, notable differences exist. Variations in the underlying support materials, *e.g.*, differences in surface roughness, pore structure, and chemical composition, may alter the effective distribution and orientation of the GO coating. Additionally, slight differences in the GO deposition process, *i.e.*, thickness variations or degree of coverage, may also lead to distinct surface energy profiles.

More precisely, the observed contact angles (M-GO < N-GO < P-GO) reflect differences in the underlying support. Mixed cellulose ester (M) has a comparatively uniform pore structure and smoother surface (see Fig. 1a–f and 2a–i), allowing more conformal GO coverage and maximized OH/COOH exposure, yielding the lowest angle (∼32° ± 5). Nylon (N) features larger, more tortuous pores that lead to slight GO aggregation and trapped air pockets (angle ∼38° ± 3), while PVDF (P) has a microfilament structure and partial hydrophobic domains that reduce effective GO coverage (angle ∼64° ± 6).

To investigate the fouling behavior, flux measurements were conducted using MQW before fouling, after BSA fouling (300 ppm), and after forward flushing to determine reversible and irreversible fouling contributions. Fig. 3b shows that the pristine M, N, and P membranes experienced a significant decline in MQW-Flux after fouling. To investigate fouling behavior and cleaning stability, two consecutive fouling–forward flushing cycles were performed using 300 ppm BSA as a model foulant. Fig. 3b shows that the pristine M, N, and P membranes experienced a pronounced decline in MQW flux after fouling, whereas the GO-coated composite membranes exhibited improved fouling resistance.

Importantly, the second fouling cycle resulted in only a marginal additional flux decline for the composite membranes, indicating minimal accumulation of irreversible fouling. As shown in Fig. 3c, M-GO and N-GO maintained consistently high flux recovery ratios (FRR) across both cycles, confirming that the majority of fouling was reversible and primarily attributed to a loosely attached cake layer rather than pore blocking. In contrast, the pristine supports exhibited progressively lower FRR values after the second cycle, suggesting a higher contribution of internal pore fouling and irreversible adsorption.

The enhanced antifouling performance of the GO-coated membranes can be attributed to increased surface hydrophilicity and the formation of a hydration layer that reduces protein adhesion. Additionally, electrostatic repulsion between negatively charged GO functional groups and BSA molecules at neutral pH further mitigates foulant deposition.

The results (see Table 1), particularly the flux recovery ratios of the M-GO membrane, are interesting and correlate strongly with the observed fouling resistivity. The higher the % FRR, the higher the fouling resistivity. Considering the data after appropriate forward flushing, M-GO showed a significantly higher % FRR (around 63%) as compared to the rest of the membranes.

**Table 1:** The complete fouling study of M, N, P, M-GO, N-GO, and P-GO membranes and their %FRR before and after forward flushing

Filter	Before forward flushing				After forward flushing			
	%FRR	%*R*_t_	%*R*_r_	%*R*_ir_	%FRR	%*R*_t_	%*R*_r_	%*R*_ir_
M	40	65	3.3	60.1	35.6	67.6	3.3	64.1
N	35.7	67.9	3.5	64.3	35.7	67.9	3.6	64.3
P	33.4	69.6	3.7	68.7	33.4	69.6	3.7	68.7
M-GO	47.3	50.1	4.3	46.6	65.7	38.3	3.9	39.7
N-GO	43.7	52.1	4.2	47.9	52	52.2	4.2	47.9
P-GO	40	55.2	3.9	49.7	51	52.7	3.7	47.5

Thus, coated membranes exhibited a significantly higher flux recovery ratio (FRR) despite foulants interacting primarily with a similar GO layer. This is because a uniform or stable GO coating on the membrane with higher FRR may reduce the likelihood of irreversible foulant adsorption. Differences in surface microstructure or charge distribution could influence the strength and reversibility of BSA adsorption.

### 3.3. Quantification of paraquat permeate using LC-ESI-QqQ-MS

After evaluating the key properties of these membranes, it was crucial to validate their actual removal performance. To accomplish this, a method was developed to assess the effluent quality by measuring paraquat concentrations after filtering a representative feed through the membranes. Using paraquat standards, a sensitive LC-ESI-QqQ-MS method was developed to quantify paraquat concentrations before and after filtration experiments for each filter. The ultimate goal was to establish a reliable multiple reaction monitoring (MRM) method, incorporating at least two MS/MS transitions. The most abundant fragment ion was selected for quantification, while the remaining ion was used for confirmation and identification purposes.

Table 2 summarizes the key parameters and highlights the significance of this method for the study. Paraquat was detected as a protonated [M + H]^+^ ion in positive mode, with retention time (RT ± SD) values demonstrating the precision and consistency of the separation and detection process. The LOD and limit of quantification (LOQ), along with the coefficient of determination (*R*^2^), attest to the method's reliability, accuracy, and ability to determine membrane removal efficiency. Notably, the LOD and LOQ values fall within acceptable ranges, ensuring accurate detection and quantification of even trace amounts of paraquat.

**Table 2:** Summary of parameters including MRM transitions for paraquat

RT ±SD	*R* ^2^	LOD (ppm)	LOQ (ppm)	MRM transitions (*m*/*z*)	
				Quantification	Identification
5.5 ± 0.01	0.99	0.04	0.12	186.8 → 171.7	186.8 → 78.3

### 3.4. Removal performance of GO membranes

The results obtained from the drug removal performance of GO composite membranes offer valuable insights into their effectiveness in addressing paraquat in water. The membranes were subjected to filtration tests using feed solutions containing paraquat at various concentrations.

**Fig. 4:**
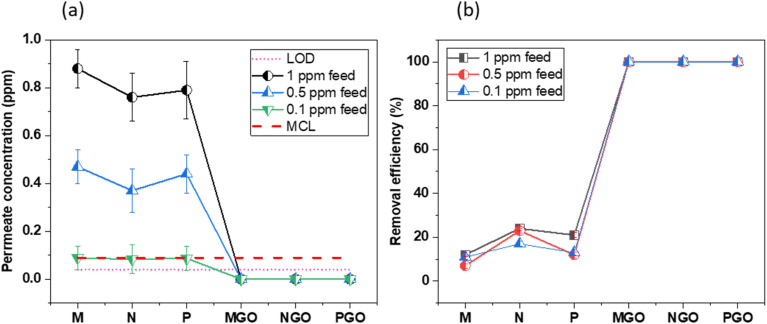
Evaluation of paraquat removal by membranes. (a): LCMS analysis of paraquat concentration in permeates of M, N, P, M-GO, N-GO, and P-GO membranes during filtration of feed concentrations ranging from 1–0.1 ppm, benchmarked against Maximum Contaminant Levels (MCLs). (b): Corresponding removal efficiencies of each membrane against various feed concentrations, demonstrating their effectiveness in removing paraquat from water.

From Fig. 4a, at a 1 ppm feed, the M membrane achieved a permeate concentration of 0.88 ppm, which is significantly above the MCL of 0.09 ppm. As the feed concentration was reduced to 0.5 ppm and 0.1 ppm, the permeate concentrations decreased to 0.08 ppm and 0.07 ppm, respectively. Notably, at the 0.5 ppm feed concentration, the permeate was still just below the MCL, indicating limited effectiveness at higher feed concentrations. The N membrane exhibited a slightly better performance with permeate concentrations of 0.76 ppm, 0.1 ppm, and 0.09 ppm for feed concentrations of 1 ppm, 0.5 ppm, and 0.1 ppm, respectively. The concentration of 0.09 ppm at a 0.1 ppm feed meets the MCL, but the membrane's performance at higher feed concentrations remains suboptimal. The P membrane demonstrated similar performance trends, with permeate concentrations of 0.79 ppm, 0.12 ppm, and 0.08 ppm for the respective feed concentrations. Like the N membrane, the P membrane's permeate at 0.1 ppm feed meets the MCL, but higher feed concentrations result in permeate levels above the acceptable limit.

In contrast, the GO composite membranes (M-GO, N-GO, P-GO) showed superior performance. For all feed concentrations tested (1 ppm, 0.5 ppm, 0.1 ppm), the GO composite membranes consistently yielded permeate concentrations below the MCL and also below the LOD of our method. This indicates the required removal of paraquat from the solution, significantly surpassing the performance of non-GO membranes and maintaining permeate concentrations well below the MCL.

The removal efficiencies for each membrane at varying feed concentrations are detailed in Fig. 4b. The efficiency trends mirror the observations from the permeate concentration data. For membrane M, the removal efficiency was 12% at a 1 ppm feed, improving to 90% and 93% at 0.5 ppm and 0.1 ppm feeds, respectively. For N Membrane, the removal efficiency was 24% at a 1 ppm feed, with increases to 77% and 91% at 0.5 ppm and 0.1 ppm feeds, respectively. The P membrane showed removal efficiencies of 21%, 88%, and 92% at the respective feed concentrations.

The GO composite membranes exhibited a 100% removal efficiency across all feed concentrations, clearly indicating their superior performance. The data demonstrate that the inclusion of GO in the composite membranes (M-GO, N-GO, P-GO) significantly enhances their capability to remove paraquat from water. This is evidenced by the complete absence of paraquat in the permeate, achieving a 100% removal efficiency even at the highest feed concentration of 1 ppm. This superior performance can be attributed to the unique properties of graphene oxide, such as its large surface area, high functionalization degree, and excellent adsorption capabilities.

In contrast, the non-GO membranes (M, N, P) showed moderate performance improvements with decreasing feed concentrations. However, they were unable to meet the MCL for paraquat at the higher feed concentrations, highlighting their limitations in handling higher contaminant loads effectively.

**Fig. 5:**
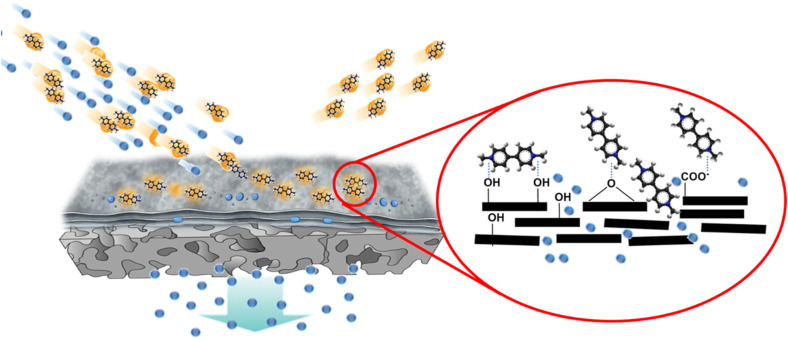
Schematic illustration of the proposed paraquat removal mechanism in GO composite membranes. Stacked GO sheets form nanochannels that allow water permeation while paraquat (dication) is retained *via* electrostatic adsorption onto negatively charged oxygen functional groups and steric confinement within interlayer galleries.

The consistently high paraquat rejection observed across all GO composite membranes indicates that both electrostatic interactions and structural confinement within the GO layer contribute to contaminant removal. A schematic representation of the proposed mechanism is shown in Fig. 5.

The deposited GO forms stacked lamellar sheets on the microporous support, generating interconnected nanochannels that permit rapid water transport while restricting contaminant passage. Paraquat, a dicationic and highly water-soluble molecule, is strongly attracted to the negatively charged oxygen-containing functional groups (–COO^−^, –OH, and epoxy groups) present on GO surfaces. This electrostatic attraction promotes the adsorption of paraquat within the interlayer galleries and on external GO sheets. Simultaneously, the confined interlayer spacing of stacked GO layers provides a steric barrier, contributing to size-based exclusion. Therefore, paraquat removal is governed by a synergistic mechanism involving electrostatic adsorption and nanochannel-mediated size exclusion.

The observed improvement in paraquat removal with increasing support roughness can be directly linked to morphology-induced effects on GO deposition and interfacial behavior. Rougher supports promote more conformal and textured GO coatings, increasing the effective surface area and the density of accessible adsorption sites.^29^ From a surface and interface science perspective, this behavior is governed by several factors. Rougher substrates typically exhibit higher apparent surface energy, which enhances wettability and facilitates uniform spreading of the aqueous GO dispersion during vacuum filtration. In addition, microscopic asperities can mechanically anchor GO flakes, improving coating adhesion and structural stability. Surface topography may also influence interfacial interactions such as hydrogen bonding and van der Waals forces between the support and GO sheets, thereby affecting coating continuity and integrity.

Together, these interfacial effects increase the exposure of negatively charged functional groups, strengthening electrostatic attraction with positively charged paraquat molecules (Fig. 5). Moreover, GO deposition enhances overall membrane hydrophilicity, promoting efficient water transport through nanochannels while maintaining strong contaminant retention. Collectively, these findings demonstrate that support morphology plays a decisive role in controlling GO layer structure, interfacial stability, and transport selectivity, ultimately dictating the balance between flux and paraquat rejection.

### 3.5. Reusability, effect of feed concentration, and simulated agricultural matrix performance

Although all three composite membranes achieved 100% paraquat removal at 1 ppm, the M-GO membrane was selected for extended evaluation due to its highest permeate flux and demonstrated structural stability over 42 h of operation.

**Fig. 6:**
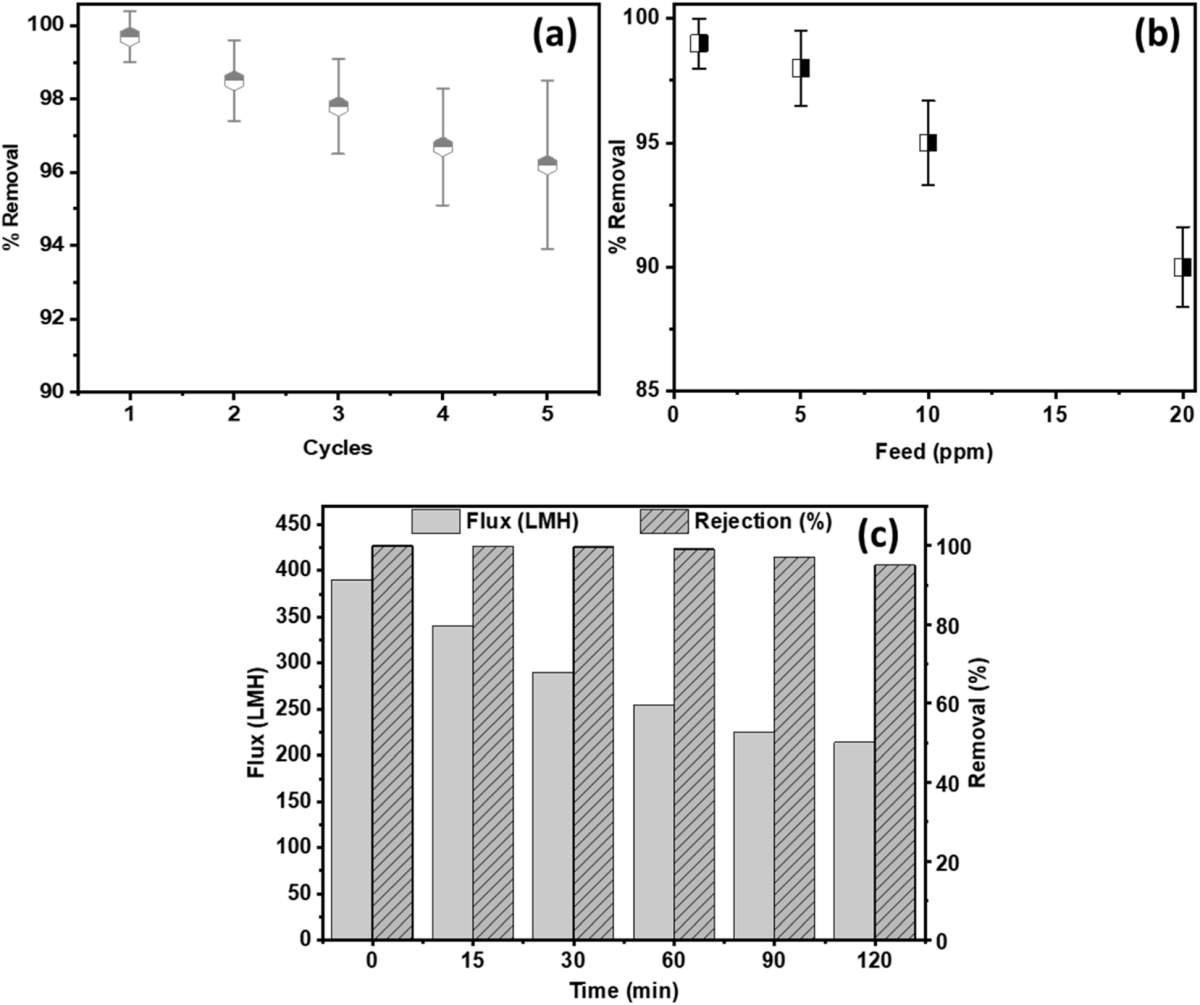
(a): Reusability performance of the M-GO membrane for paraquat removal using a 1 ppm feed during five consecutive filtration cycles at 0.2 bar. (b): Paraquat rejection as a function of feed concentration (1–20 ppm). (c): Flux and paraquat rejection as a function of time during filtration in a simulated agricultural water matrix (5 ppm paraquat, 100 mM NaCl, 10 ppm HA), TMP = 0.2 bar, area = 12 cm^2^.

Reusability was assessed through five consecutive filtration cycles using a 1 ppm paraquat feed at 0.2 bar TMP. Each cycle consisted of 1 h of steady-state filtration, followed by rinsing with MQW without chemical cleaning. As shown in Fig. 6a, the removal efficiency remained ≥ 95% throughout all five cycles, with only a marginal decline in flux, confirming excellent operational stability and intrinsic cleanability of the GO coating. Furthermore, no GO mass was observed in the filtrate, which confirms strong adhesion between the GO layer and the polymer support.

To further evaluate robustness beyond trace conditions, the M-GO membrane was tested at higher paraquat feed concentrations (5, 10, and 20 ppm) under identical operating conditions (0.2 bar). As shown in Fig. 6b, paraquat rejection remained high, decreasing gradually from complete removal at 1 ppm to approximately 98%, 95%, and 90% at 5, 10, and 20 ppm, respectively. The modest reduction in rejection at elevated concentrations can be attributed to concentration polarization effects and partial occupation of available adsorption sites on the GO surface. Notably, even at 20 ppm, the membrane maintained > 90% removal, demonstrating strong tolerance to increased contaminant loading.

To simulate more realistic agricultural discharge conditions, the optimized membrane was further evaluated using a model matrix containing paraquat (5 ppm), NaCl (100 mM), and humic acid (10 ppm) as a representative natural organic matter surrogate. Under these conditions, a progressive flux decline was observed over 120 min of operation (Fig. 6c), stabilizing at approximately 55–60% of the initial flux. This behavior is consistent with humic-acid-induced fouling reported for charged membranes and reflects the combined effects of organic adsorption, pore blocking, and electrical double-layer compression at elevated ionic strength. Importantly, paraquat rejection remained above 90% throughout the experiment, indicating that electrostatic interactions between negatively charged GO functional groups (–COOH, –OH) and the cationic paraquat molecules remain dominant even in the presence of competing ions and natural organic matter.

Overall, these results demonstrate that the optimized GO composite membrane maintains high separation performance under elevated contaminant concentrations and in complex aqueous matrices. While validation with real agricultural discharge is warranted in future work, the present findings confirm the membrane's strong potential for practical water treatment applications beyond idealized laboratory conditions.

### 3.6. Comparative evaluation with literature

To contextualize our findings, we compared the performance of our GO-coated microporous membranes (M-GO, N-GO, and P-GO) with previously reported GO-based membrane systems used for pesticide and organic contaminant removal. Table 3 summarizes key parameters, including membrane support, GO deposition method, target contaminants, operating pressure, flux, and removal efficiency. In this study, the GO composite membranes achieved complete paraquat rejection (100%) over a feed concentration range of 0.1–1.0 ppm at a very low operating pressure of 0.2 bar, while maintaining high permeate flux in the range of approximately 200–420 L m^−2^ h^−1^ (abbreviated as LMH). These values compare favorably with other GO-modified systems reported for pesticide removal, many of which operate at higher pressures or exhibit lower flux or partial rejection.

**Table 3:** Performance comparison of GO-coated microporous polymer membranes for contaminant removal, highlighting membrane type, operating conditions, and removal efficiency

Ref.	Membrane support & material	Coating method	Target contaminants	Operating pressure (bar)	Flux (LMH)	Removal efficiency (%)
This study	Mixed cellulose ester, nylon, PVDF (0.45 µm)	Vacuum-assisted filtration (1 bar) of GO dispersion (0.42 mg cm^−2^)	Paraquat	0.20	∼200–420	100 (0.1–1.0 ppm feed)
22	Nylon (0.45 µm)	Vacuum filtration (0.1 bar) of GO (0.04–0.06 mg cm^−2^)	2-Naphthol, dyes	2.0	40–60	64–96 (UV dependent)
23	PVDF microfiltration membrane	GO-chitosan layer *via* vacuum filtration (citric acid assisted)	Rhodamine B, Methylene Blue	∼0.1–0.5	Not given	69.1 (MB), 96.0 (RB)
24	Macroporous ceramic UF (with chitosan interlayer)	Dip coating of chitosan followed by GO	Atrazine	∼0.1–1.0	Not given	>95
30	Polysulfone (UF) mixed-matrix membranes	GO/TiO_2_ nanocomposite embedded *via* phase inversion	Glyphosate, 2,4-d, Trifluralin, Butachlor	1.0	7.3–326	50–70
31	NF270 (commercial NF)	Pristine	Atrazine	5–10 bar	20–50	>95%
32	NF70 (commercial NF)	Pristine	Multiple pesticides	< 10 bar	Not given	>99%
33	SW30XLE (RO)	Pristine	Pesticides	10–20 bar	0.5–0.7	>95%

Additionally, we contextualized our results with literature-reported performance of commercial nanofiltration (NF) and reverse osmosis (RO) membranes. For example, the commercial NF270 membrane has been reported to achieve >95% atrazine rejection at operating pressures of 5–10 bar, with flux values typically in the range of 20–50 LMH. Similarly, NF70 membranes have demonstrated >99% rejection of multiple pesticides under pressures below 10 bar, while RO membranes such as SW30XLE have achieved >95% pesticide removal at significantly higher pressures (10–20 bar) but with substantially lower flux (0.5–0.7 LMH). These comparisons indicate that although commercial NF/RO technologies can achieve high pesticide rejection, they generally require much higher TMP and deliver lower flux compared to our GO composite membranes, which achieve 100% paraquat removal at only 0.2 bar.

## 4 Conclusion

This work's novelty is a controlled head-to-head comparison at identical GO mass loading (0.42 mg cm^−2^) across three low-cost microporous supports, revealing support-dependent GO morphology–flux–rejection relationships and enabling high-flux paraquat removal at very low pressure (0.2 bar). By maintaining constant fabrication and operating conditions, this study isolates the decisive role of support surface roughness and pore architecture in governing GO layer formation, accessible adsorption functionality, antifouling behavior, and the resulting permeability–selectivity balance.

The results demonstrate that the M-GO membrane achieved the highest steady-state water flux (425 ± 10 L m^−2^ h^−1^) while maintaining near-complete paraquat rejection under low TMP. Extended operation (42 h), repeated reuse cycles, and testing under a simulated agricultural matrix further confirm the structural stability and robustness of the optimized composite membrane.

Paraquat was selected as the model contaminant due to its high environmental relevance, very high water solubility, and strong cationic nature. Its high solubility enhances its mobility in agricultural runoff, increasing the likelihood of transport into surface and groundwater systems. These characteristics make paraquat a particularly challenging and environmentally significant target, while also enabling clear assessment of electrostatic interactions between the dicationic pesticide and negatively charged GO functional groups.

While this study successfully demonstrates the high paraquat removal efficiency and flux performance of GO composite membranes, further investigation is required to assess their behavior in complex, real-world water matrices containing multiple contaminants, natural organic matter, and competing ions. In addition, long-term operational stability under variable pH and TMP conditions warrants deeper evaluation. Evaluation of additional pesticide classes, including neutral and anionic species, will be pursued in future studies to broaden the applicability of this membrane platform.

Importantly, our findings highlight the critical role of support surface roughness in dictating the morphology, continuity, and performance of GO-coated membranes. Controlled modulation of surface roughness offers a practical strategy to tailor membrane flux and contaminant rejection. However, excessive roughness may promote non-uniform GO deposition, leading to structural defects and performance variability. Future research should therefore focus on quantitatively correlating support roughness parameters with GO layer structure and transport properties to establish design guidelines for optimized composite membrane fabrication.

## Consent to publish

All authors have read and agreed to the published version of the manuscript.

## Author contributions

Syed Sibt-e-Hassan (conceptualization, methodology, investigation, data curation, formal analysis, writing – original draft). Nurmeen Adil (investigation, validation, data curation). Syed Ghulam Musharraf (supervision, project administration, resource, validation).

## Conflicts of interest

The authors declare no conflict of interest.

## Supplementary Material

RA-016-D6RA00505E-s001

## Data Availability

All data supporting this study are included in the article and its supplementary information (SI). No additional data are associated with this work. Supplementary information is available. See DOI: https://doi.org/10.1039/d6ra00505e.
