# Supplementary material for: Screening of GO-coated microporous polymeric filters for efficient paraquat removal: effect of support surface on membrane roughness and flux stability
Source: RSC Adv. 2026 Mar 20;16(17):15802–13. doi: 10.1039/d6ra00505e (PMC13003536; doi:10.1039/d6ra00505e)
Supplement: RA-016-D6RA00505E-s001 [file RA-016-D6RA00505E-s001.pdf]

Supplementary Information (SI) for RSC Advances.
This journal is © The Royal Society of Chemistry 2026

Screening of GO-Coated Microporous Polymeric Filters for Efficient Paraquat Removal: Effect of Support Surface on Membrane Roughness and Flux Stability

Syed Sibt-e-Hassan[a], Nurmeen Adil[a,b], Yan Wang[c], Syed Ghulam Musharraf[a]*

[a]HEJ Research Institute of Chemistry, International Center for Chemical and Biological Sciences, University of Karachi, Karachi-75270 Pakistan

[b]Swedish Metabolomics Centre, Swedish University of Agricultural Sciences, Umeå, Sweden

[c]School of Chemistry and Pharmaceutical Sciences, Guangxi Normal University, Guilin-541004, China

*Corresponding author. Tel.: +92 21 34824924-5; 34819010; Telefax: (+ 92-21) 4819018-9
E-mail address: musharraf1977@yahoo.com, musharraf@iccs.edu

Characterization of GO

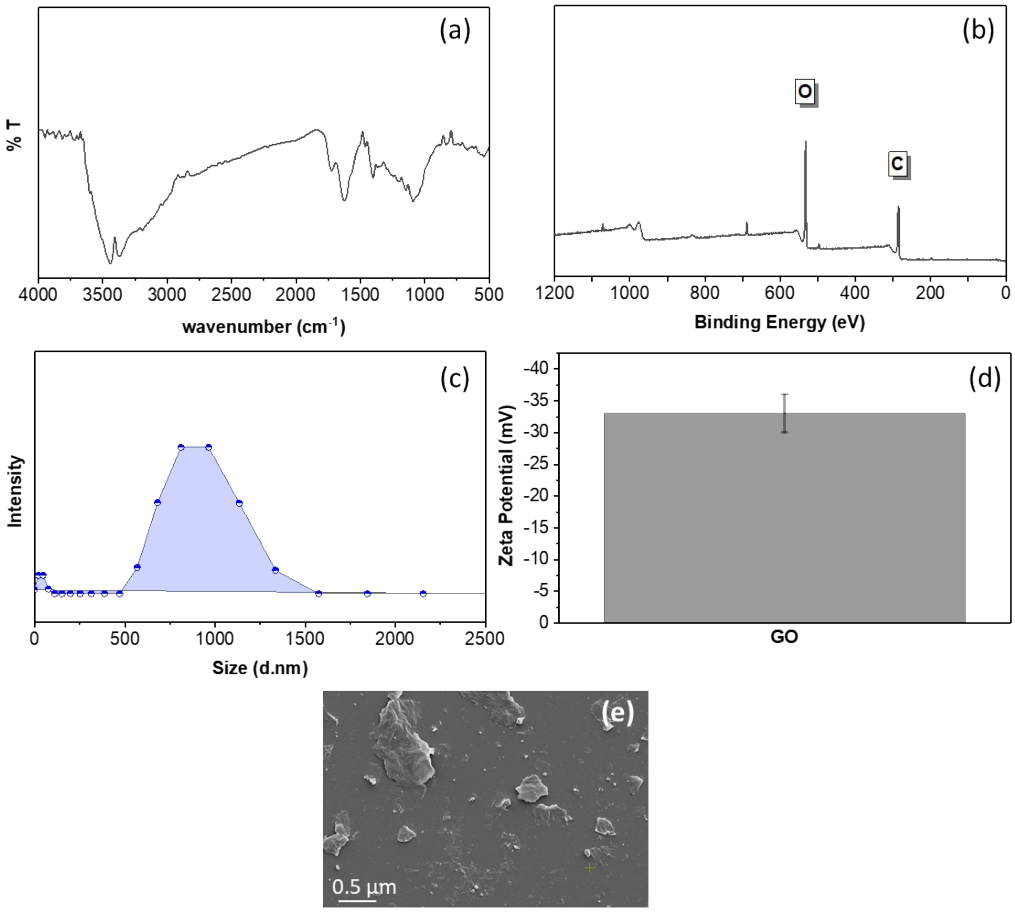

Figure S1. *Figure S1. Characterization of synthesized graphene oxide (GO): (a) FT-IR spectrum, (b) XPS survey spectrum, (c) DLS particle size distribution, and (d) SEM image of GO sheets (scale bar: 0.5 µm).*

The structural and physicochemical properties of the synthesized graphene oxide (GO) were confirmed using FT-IR, XPS, DLS, zeta potential analysis, and SEM (detailed spectra and analyses are provided in the Supporting Information, Figures S1).

FT-IR analysis confirmed the presence of characteristic oxygen-containing functional groups, including O–H stretching (~3400 cm^{-1}), C=O stretching (~1720 cm^{-1}), C=C skeletal vibrations (~1620 cm^{-1}), and C–O stretching (~1050–1220 cm^{-1}), indicating successful oxidation of

graphite. XPS survey spectra revealed dominant C and O peaks, and high-resolution C 1s deconvolution confirmed the presence of C–C/C=C, C–O, C=O, and O–C=O functionalities, consistent with typical GO chemistry

Dynamic light scattering showed a broad flake size distribution in aqueous dispersion, while zeta potential measurements indicated a strongly negative surface charge, reflecting good colloidal stability and the presence of ionizable oxygenated groups. SEM micrographs revealed thin, wrinkled GO sheets with lateral dimensions in the sub-micron to micron range.

Characterization of GO Composite Membranes

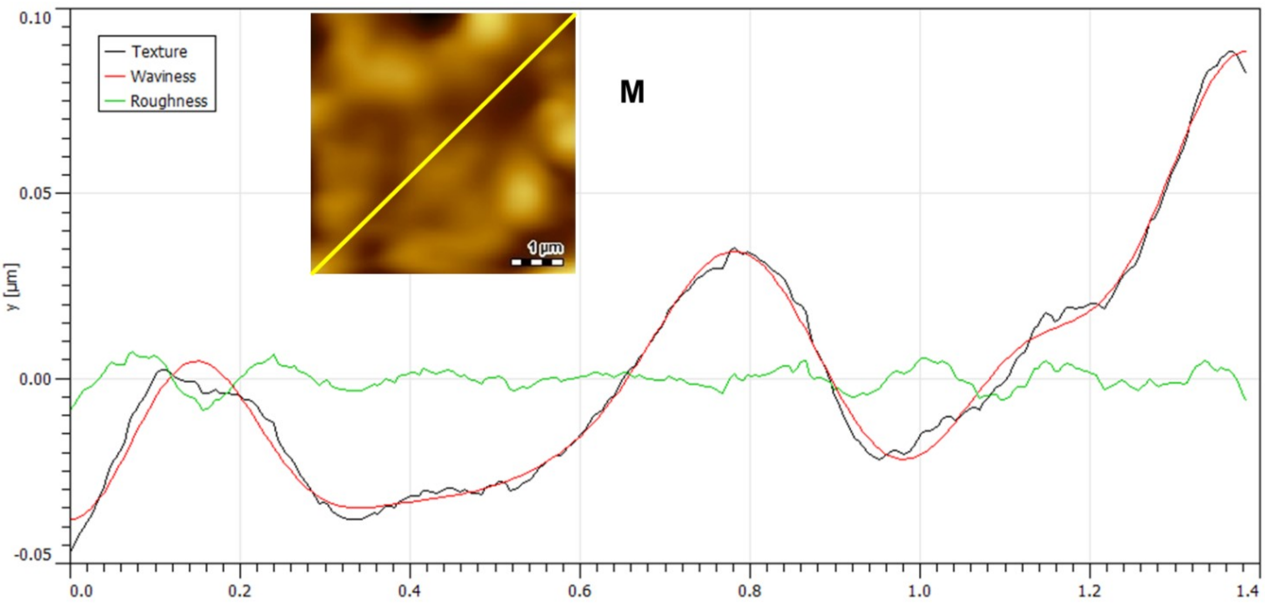

Figure S2. *AFM surface morphology of the pristine Mixed Cellulose Easter (M) filter and corresponding roughness profile. The inserted AFM image, with the yellow trace indicating the line along which roughness was measured. The graph presents the surface-roughness profile extracted along that line using Gwyddion software.*

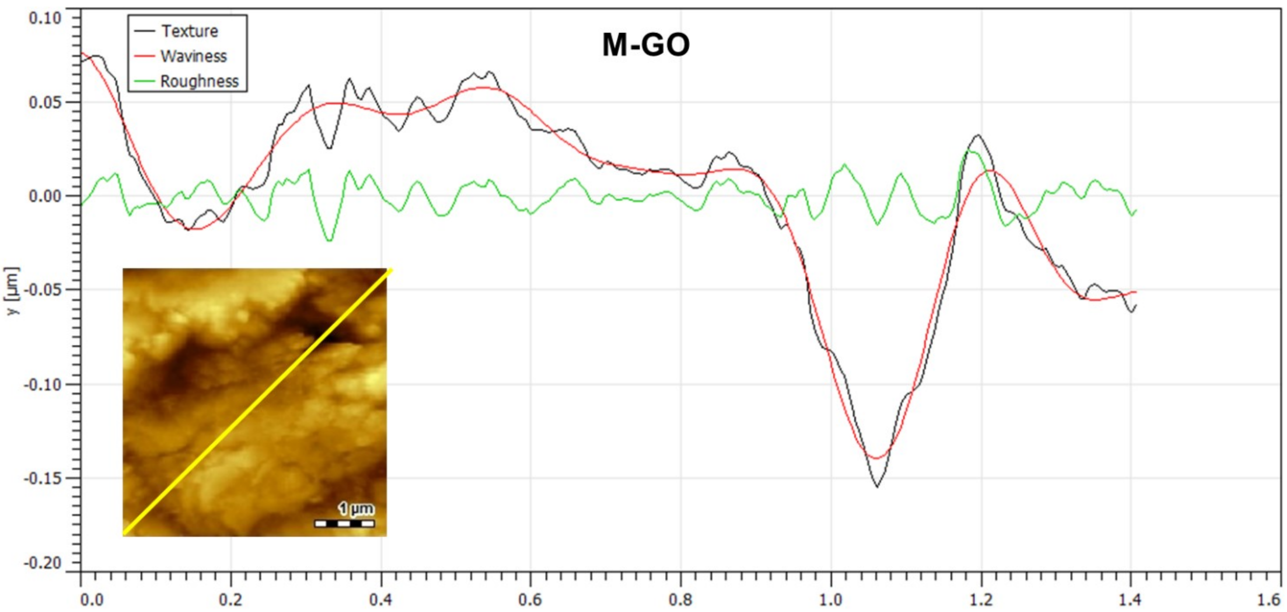

Figure S3. *AFM surface morphology of the M-GO composite membrane and corresponding roughness profile. The inserted AFM image, with the yellow trace indicating the line along which roughness was measured. The graph presents the surface-roughness profile extracted along that line using Gwyddion software.*

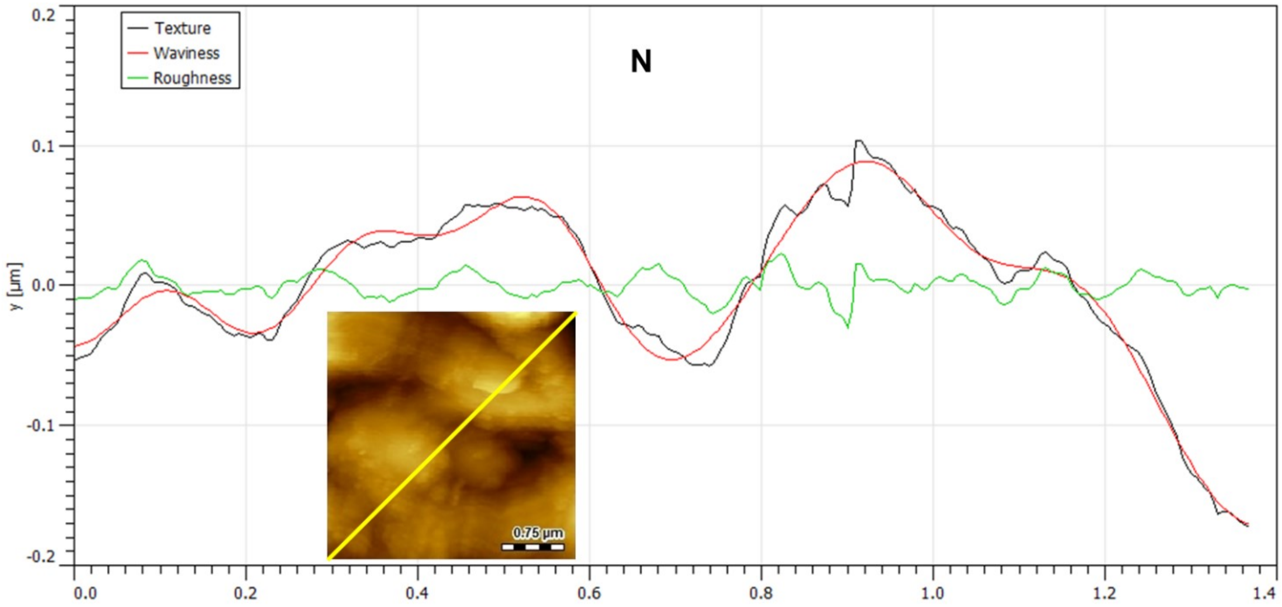

Figure S4. *AFM surface morphology of the pristine Nylon (N) filter and corresponding roughness profile. The inserted AFM image, with the yellow trace indicating the line along which roughness was measured. The graph presents the surface-roughness profile extracted along that line using Gwyddion software.*

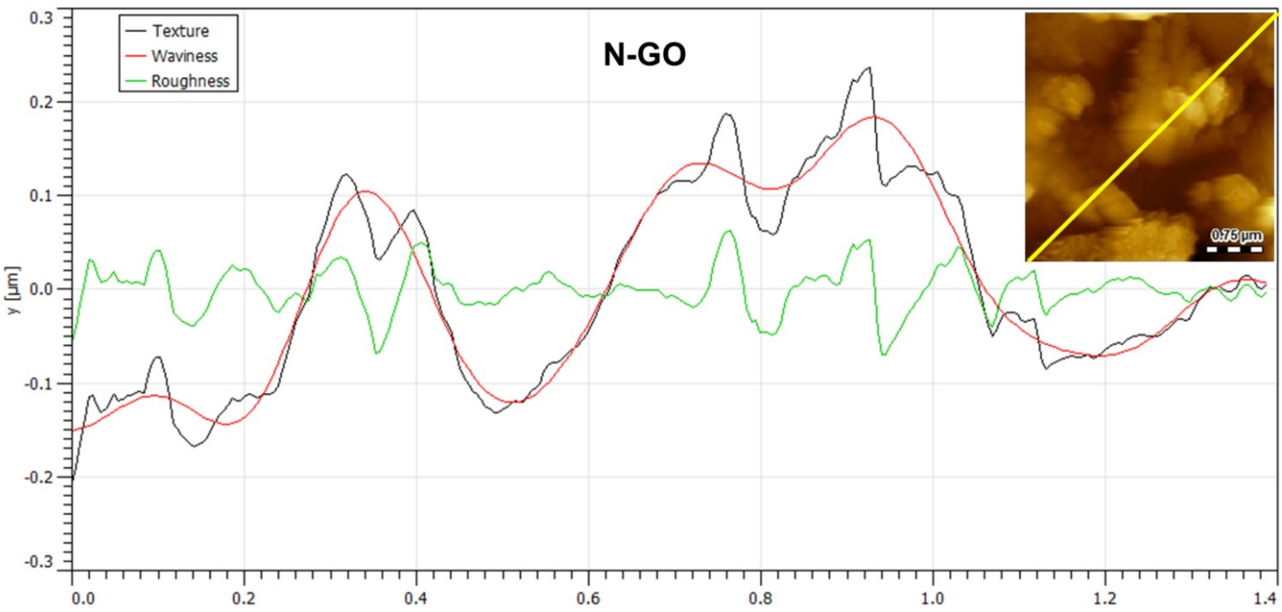

Figure S5. *AFM surface morphology of the N-GO composite membrane and corresponding roughness profile. The inserted AFM image, with the yellow trace indicating the line along which roughness was measured. The graph presents the surface-roughness profile extracted along that line using Gwyddion software.*

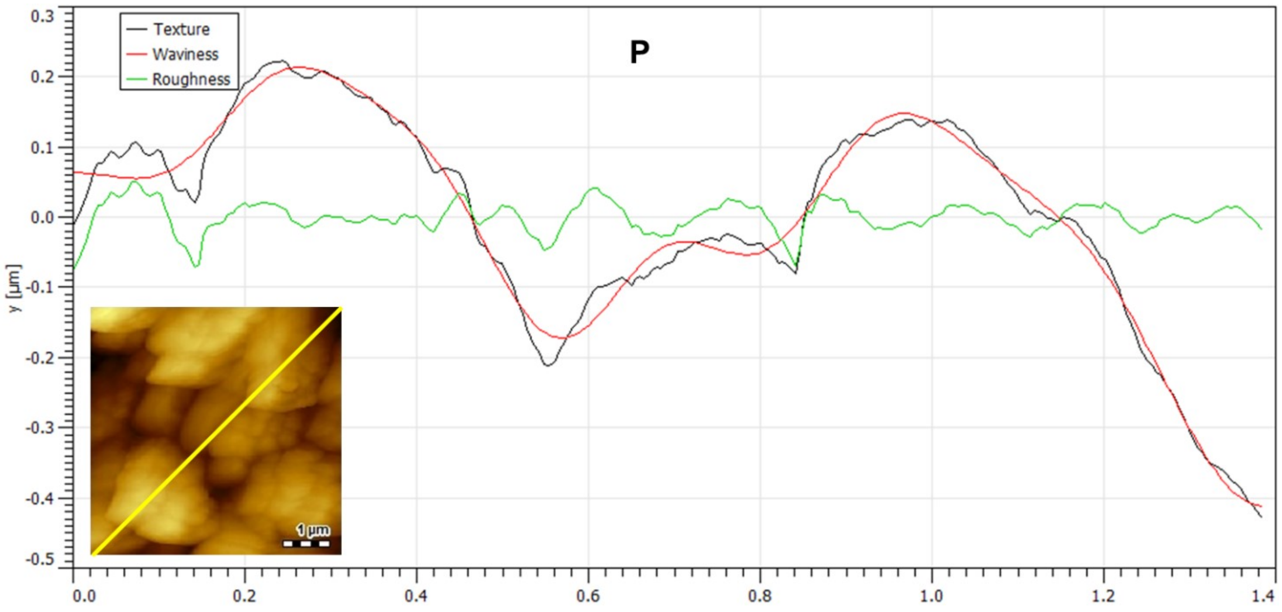

Figure S6. *AFM surface morphology of the pristine PVDF (P) filter and corresponding roughness profile. The inserted AFM image, with the yellow trace indicating the line along*

which roughness was measured. The graph presents the surface-roughness profile extracted along that line using Gwyddion software.

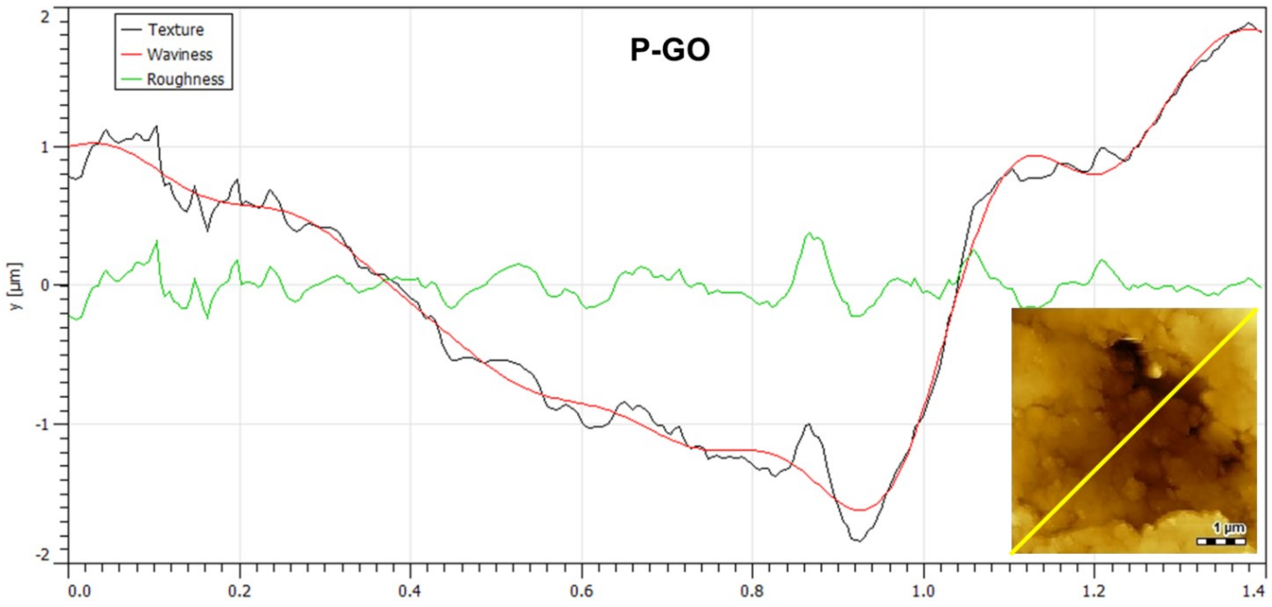

Figure S7. *AFM surface morphology of the P-GO composite membrane and corresponding roughness profile. The inserted AFM image, with the yellow trace indicating the line along which roughness was measured. The graph presents the surface-roughness profile extracted along that line using Gwyddion software.*

Porosity and Water Uptake Study

For water uptake analysis, 1 g membrane samples were sealed with epoxy, dried at 40 °C to constant weight, and soaked in Milli-Q water for 24 h. After blotting surface moisture, wet weight (Ww) was recorded. Samples were then dried to obtain dry weight (Wd). Water uptake (%) was calculated as:

$$\% \text{ Water Uptake} = (W_w - W_d) / W_w \times 100$$

Membrane porosity (ε) was determined using:

$$\varepsilon (\%) = (W_w - W_d) / (A \times l \times \rho) \times 100$$

Where A is membrane area (cm^2), l is thickness (cm), and ρ is water density (0.998 g/cm^3).

Table S1. Gravimetric porosity of the membranes. Golden columns represent pristine membranes, while blue columns correspond to GO composite membranes.

0.4 um Pristine filters	Gravimetric Porosity (%)	Composite Membranes	Gravimetric Porosity (%)
Mixed Cellulose Ester (M)	75–82 %	***M-GO***	60-69%
Nylon (N)	72–78 %	***N-GO***	57-65%
PVDF (P)	58–68 %	***P-GO***	43-47%

These values reflect progressive reduction due to GO deposition (~10 % drop for M-GO, ~15 % for N-GO, ~20 % for P-GO), consistent with literature observations of GO-induced pore blocking and layer thickening upon rougher supports.

The smooth, uniform MCE allows conformal GO coverage with limited aggregation resulting in ~10 % porosity loss for thin GO layers on smooth supports. Tortuous nylon pores trap GO, causing ~15 % reduction, showing GO-induced flux decline correlating with pore blocking in GO-polymer composites. PVDF's high roughness and hydrophobic domains promote GO aggregation and thick layer formation, yielding ~20 % reduction.

Post-Coating Morphological Observations

Due to the highly flexible and ductile nature of the polymer support, obtaining a clean cross-sectional SEM image of the bilayer membrane is challenging. Several attempts were made using sharp blades as well as cryogenic fracturing after immersion in liquid nitrogen; however, the substrate did not produce a well-defined fracture surface suitable for cross-sectional analysis.

To address this limitation, the coated membranes were immersed in liquid nitrogen and mechanically stressed by bending from opposite sides while submerged. This approach selectively fractured the top GO layer, allowing it to be gently peeled off and examined in free-standing form. The SEM images presented below (see ***Figure S8***) show the detached GO layer, confirming the formation of a continuous and compact coating.

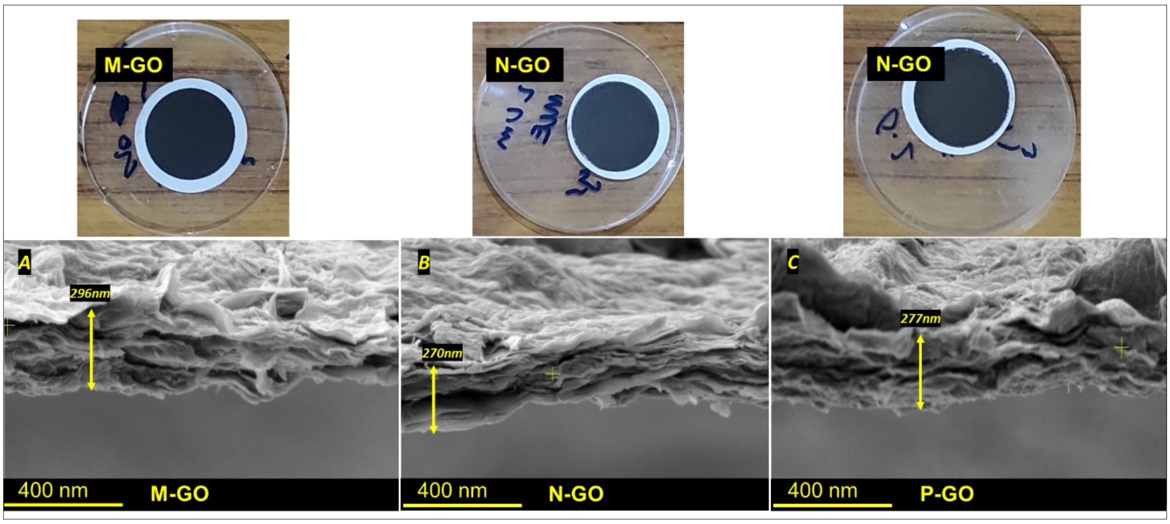

Figure S8. *Photographs of M-GO, N-GO, and P-GO composite membranes, along with their cross-sectional SEM images obtained after careful peeling following liquid nitrogen immersion.*

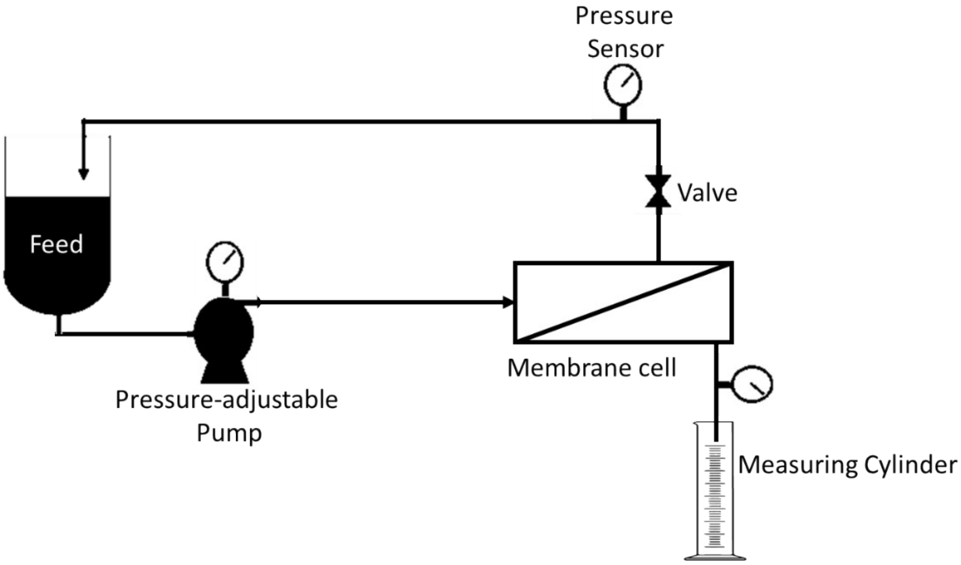

Figure S9. *Schematic illustration of the experimental filtration setup used for performance evaluation, showing feed reservoir, pressure control, pump system, and membrane module arrangement.*
